# 

**Published:** 1975-01

**Authors:** 


Bristol Medico-Chirurgical Journal Vol. 90 (i) No. 334

### Contents

Primary Hodgkin's disease of the rectum ... 1
R. Salm and C. M. Vickery

Tumour-to-tumour Metastasis ... ... ... 5

R. Salm

An Atypical case of Spontaneous Rupture of the Oesophagus ... ... ... ... ... 9
A. A. J. Barros D'sa

New Voices for old ... ... ... ... 11

Nigel Edwards

F. Bailey & Son Ltd., Dursley, Glos.

